# Antimicrobial Activity of a Synthetic Brevibacillin Analog Against Multidrug-Resistant *Campylobacter* spp.

**DOI:** 10.3390/ijms26104657

**Published:** 2025-05-13

**Authors:** Khaled Abdallah, Omar Fliss, Nguyen Phuong Pham, Louis David Guay, Hélène Gingras, Chantal Godin, Philippe Leprohon, Eric Biron, Ismail Fliss, Marc Ouellette

**Affiliations:** 1 Centre de Recherche en Infectiologie du Centre de Recherche du CHU Québec et Département de Microbiologie, Infectiologie et Immunologie, Faculté de Médecine, Université Laval, Québec City, QC G1V 4G2, Canada; khaled.abdallah.1@ulaval.ca (K.A.); nguyen-phuong.pham@crchudequebec.ulaval.ca (N.P.P.);; 2 Département des Sciences des Aliments et de Nutrition, Université Laval, Québec City, QC G1V 0E8, Canada; 3 Faculté des Sciences de Tunis, Université de Tunis El Manar, Tunis 2092, Tunisia; 4 Faculté de Pharmacie, Université Laval et Laboratoire de Chimie Médicale, Centre de Recherche du CHU de Québec-Université Laval, Québec City, QC G1V 4G2, Canadaeric.biron@pha.ulaval.ca (E.B.)

**Keywords:** *Campylobacter* spp., brevibacillin, antibiotics, WGS, multidrug resistance

## Abstract

*Campylobacter* spp. is one of the most prevalent causes of zoonotic foodborne infections associated with diarrhea in humans. The growing threat of antibiotic resistance calls for innovative approaches. The antimicrobial lipopeptide brevibacillin produced by *Brevibacillus laterosporus* and its synthetic analog brevibacillin Thr1 showed promising activity against *Salmonella* and *E. coli*. The latter is a 1602.13 Da positively charged (+3) synthetic peptide of 13 residues that showed reduced cytotoxicity (IC_50_ of 32.2 µg/mL against Caco-2 cells) and hemolytic activity (1.2% hemolysis at 128 µg/mL) compared to the native peptide. It contains an N-terminal L-isoleucic fatty acid chain and four non-proteinogenic amino acids and ends with valinol at its C-terminus. One key structural modification is the substitution of α,β-dehydrobutyric acid with threonine. We investigated the antimicrobial potential of the synthetic brevibacillin Thr1 analog against a collection of 44 clinical *Campylobacter* spp. that were obtained from two reference laboratories. Susceptibility testing revealed marked resistance to ciprofloxacin, tetracycline, and ampicillin among the strains, with more than half expressing a multidrug-resistant phenotype. The genomes of the 44 strains were sequenced to study the genes responsible for their antimicrobial resistance. Tetracycline resistance was associated with *tet*(*O*), ciprofloxacin resistance with mutations in *gyrA* and regulatory sequences modulating the expression of an efflux system, and aminoglycoside resistance with genes of the *aph* family. The brevibacillin Thr1 analog was produced by chemical synthesis, and evaluation of its activity against a subset of clinical strains by microdilution revealed minimum inhibitory concentration and minimum bactericidal concentration ranging from 8 µg/mL to 64 µg/mL. The peptide was active against multidrug-resistant isolates with a bactericidal effect. Of note, despite numerous attempts, it proved impossible to select *Campylobacter* spp. for resistance to the brevibacillin Thr1 analog. These results underline the potential of lipopeptides, notably brevibacillin, as antimicrobial alternatives against antibiotic-resistant *Campylobacter* bacterial infections.

## 1. Introduction

Antimicrobial resistance is a global public health concern that has been attributed to several factors, including the misuse of antibiotics in human medicine and factory farming, inadequate hygiene, sanitation, and the ineffective prevention and control of infections in healthcare settings [1]. The emergence and dissemination of antibiotic-resistant bacteria have serious consequences for the treatment of infectious diseases, as well as for patient outcomes and overall healthcare costs [2]. If left unchecked, this trend is estimated to lead to a future where many common bacterial infections will become difficult to treat. Currently, antibiotic-resistant infections cause at least 700,000 deaths each year, and in the absence of coordinated and effective actions, this phenomenon will lead to the death of 10 million people per year by 2050 [3]. The genus *Campylobacter* is listed as one that causes the most food-borne infections globally, even in high-income countries like the USA and the European Union where the costs are estimated to be around USD 2.4 billion a year [4,5]. In Canada, campylobacteriosis is consistently the most commonly reported enteric disease, with an estimated incidence of around 27 cases per 100,000 population [6]. This rate is significantly higher than those of other major enteric pathogens like *Salmonella* (~15 cases per 100,000 population) or *Escherichia coli* O157:H7 (<5 cases per 100,000 population) [7]. *Listeria monocytogenes*, with its potential for severe health outcomes and high case-fatality rate, accounts for only a small portion of enteric illnesses in the country, with reported incidence ranging from 0.2 to 1 case per 100,000 population [8]. Typical symptoms of enteric infections caused by *Campylobacter* include diarrhea, fever, abdominal cramps, nausea, and vomiting. The illness usually resolves within a week, although high-risk groups like neonates, the elderly, and immunocompromised individuals can experience serious complications such as meningitis [9], bacteremia, septicemia, or endocarditis [10,11,12]). Antibiotics are only recommended for severe or prolonged cases. Erythromycin, ciprofloxacin, and azithromycin are the drugs of choice, although resistance is now frequent [13]. *Campylobacter* is primarily commensal in birds [14] and it is estimated that poultry is a reservoir for 50–80% of human infections [15]. *Campylobacter* infection of poultry meat is indeed a major concern for food safety [16]. Cattle also act as a major reservoir and could be responsible for 20–30% of *Campylobacter* infections in humans.

Various strategies were proposed to overcome *Campylobacter* infections. One of the promising avenues involves the use of antimicrobial peptides (AMPs) [17]. AMPs represent a diverse class of molecules that are prevalent in a wide range of organisms including microorganisms, insects, plants, and vertebrates. They serve an important role in protecting against a wide range of infectious pathogens by inhibiting bacteria, fungi, parasites, and viruses. Noteworthy instances of such peptides include humimycin produced by *Rhodococcus equi*, brevibacillin from *Brevibacillus laterosporus*, as well as bacteriocins, a prominent family of AMPs secreted by the *Enterobacteriaceae* family [18,19,20]. AMPs exert their antimicrobial action through a variety of mechanisms, including membrane interactions, inhibition of essential intracellular processes, and modulation of the immune response. The antimicrobial efficacy is largely determined by their amphipathic helical structure or β-sheet conformation, as well as the distribution of cationic charges, which are critical for their interaction with microbial targets [21,22]. AMPs contribute to the intricate dynamics of microbial communities and have potential applications in food preservation, probiotics, and the development of novel antimicrobial agents. Among AMPs, lipopeptides are a class of molecules composed of hydrophilic peptides bonded to hydrophobic fatty acids. These peptides are biosynthesized through secondary metabolite pathways and they are known for their ability to target membranes, act as drug carriers adsorbed to cells, and support the immune system and for their antimicrobial properties. Several studies were performed to evaluate the potential of lipopetides as antibacterial agents in medical and agri-food fields [23,24]. One of the most promising lipopeptides, brevibacillin, is produced by *Brevibacillus laterosporus* and consists of 13 amino acids with an N-terminal C6-fatty acid chain. It has a molecular mass of 1583 Da and contains three modified amino acid residues: valinol, D-ornithine, and α,β-didehydrobutyric acid (Dhb) [25].

Significant antimicrobial activity was reported for brevibacillin and its analogs against Gram-positive and some Gram-negative bacteria implicated in foodborne disease but not yet against a collection of *Campylobacter*. Brevibacillin isolated from *B. laterosporus* OSY-I1 exhibits substantial inhibition of methicillin-resistant *Staphylococcus aureus* (MRSA), *Listeria monocytogenes*, *Escherichia coli*, and *Pseudomonas aeruginosa* [25,26]. The clinical use of brevibacillin is limited by its hemolytic activity, its high cost, and the low yields of production by fermentation. However, a chemical synthesis approach can be used to produce several brevibacillin analogs of improved efficacy. This study presents the phenotypic and genomic characterization of a collection of multidrug-resistant *Campylobacter* strains and the evaluation of the potential of the synthetic brevibacillin Thr1 analog against these strains.

## 2. Results

### 2.1. Antimicrobial Susceptibility Testing

Prior to testing the activity of brevibacillin we first characterized our collection of 44 *Campylobacter* isolates phenotypically and at the genomic level. Their susceptibility to seven antibiotics was determined using the disc diffusion method. Non-susceptibility to ampicillin (i.e., resistant and intermediate phenotypes) was widespread (90.9%) (Table S1). Non-susceptibility to ciprofloxacin and tetracycline was observed for 70.45% and 59.09% of the strains, respectively. Twenty-four strains were resistant to at least three antibiotics (Table S1) and were thus considered as multidrug resistant. Only one strain was resistant to erythromycin and no resistance to ertapenem was observed (Table S1).

**Figure 1:**
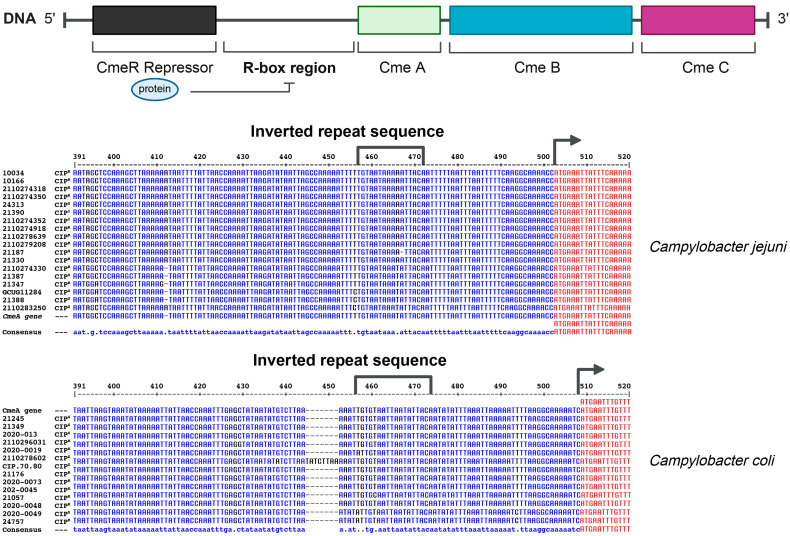
Genomic organization and features of CmeR-CmeA intergenic region. The CmeABC operon is composed of three genes, a repressor, and a regulatory region that is negatively regulated by CmeR by binding to a 16-base-pair inverted repeat sequence in the promoter region (**top**). In our strains, we have identified point mutations that have been reported in the literature as causing overexpression of *CmeABC*. Alignment of the CmeR+CmeA region of *Campylobacter jejuni* strains (**middle**). Alignment of the CmeR+CmeA region of *Campylobacter coli* strains (**bottom**). Arrows indicate the start of the *cmeA* coding region, whose nucleotides are colored in red. Nucleotides in blue are from the R-box region upstream of *cmeA*.

The genome of the *Campylobacter* isolates was sequenced in the search for genes that could explain their resistance (or lack of) to the tested antibiotics. The disc diffusion method revealed ciprofloxacin sensitive, intermediate, and resistant isolates. The intermediate isolates were often in contrast to what was suggested by the genomic data. Indeed, the genome sequence of these strains had no known mutations that could explain an intermediate phenotype. For a set of selected strains with defined genotypes we then determined the MIC values, which were in much better agreement with the genomic data (Table 1). The presence of the T86I mutation in the ciprofloxacin target GyrA was present in all the resistant isolates of *C. coli* (*n* = 9), *C. jejuni* (*n* = 8), and *C. fetus* (*n* = 1). This mutation is associated with a MIC of 8 µg/mL (Table 1). Seven *C. jejuni* strains non-susceptible to ciprofloxacin with no mutations in *gyrA* instead had point mutations in a 16-base-pair inverted repeat located within the regulatory region of the *Campylobacter cmeR-cmeABC* efflux pump (Figure 1, Table 1). These cells had a MIC of 4 µg/mL to ciprofloxacin (Table 1). Four *C. jejuni* strains and one *C. coli* had mutations both in *gyrA* and in the 16 bp inverted repeat of the *cmeR-cmeABC* regulatory region (Figure 1). These cells were not more resistant (8 µg/mL), however, than cells with solely the T86I mutation in GyrA (Table 1). The ciprofloxacin MICs were concordant with the genome data for all isolates except one, *C. jejuni* strain 21387, which had a ciprofloxacin MIC of 4 µg/mL but with no mutation that could explain its phenotype. In *C. lari*, *gyrA* was also mutated in four strains but, in this case, and in contrast to *C. jejuni* and *C. coli*, it was a T86V mutation associated with a ciprofloxacin MIC of 4 g/mL (Table 1).

Twenty-six bacteria were resistant to tetracycline, as determined with the disc diffusion method (Table 1). The *tet*(*O*) gene was found in only 18 strains according to the genome sequence data (Table 1). Eight strains were thus resistant to tetracycline, but their sequencing failed to detect *tet*(*O*). This is surprising as *tet*(*O*) is the most common tetracycline resistance gene in *Campylobacter* [27]. We thus carried out a PCR assay to test whether *tet*(*O*) was present in these eight strains and could have been missed through our sequence analysis. PCRs indeed confirmed that *tet*(*O*) was present in these eight strains (Table 1, Figure S1). It should be noted that, in 8 of the 18 strains where *tet*(*O*) was detected by genome sequencing, we also detected the presence of the pTet plasmid [28] with various Refseq sequence types (Table S2). We could not detect this plasmid in the 10 other strains positive for *tet*(*O*) by genome sequencing, nor in those only positive for *tet*(*O*) by PCR (Table S2). 

**Table 1:** Antibiotic susceptibility status and genetic determinants of resistance detected in *Campylobacter* spp.

Strains		Ciprofloxacin				Tetracycline				Gentamycin			Ampicillin		
		Disc ^a^	MIC ^b^	*gyrA* ^c^	R-box ^d^	Disc ^a^	MIC ^b^	*tet*(*O*) ^e^		Disc ^a^	MIC ^b^	Genotype ^f^	Disc ^a^	MIC ^b^	bla _OXA-61_^g^
								NGS	PCR						
***C. jejuni***															
**1**	CCUG11284	S	1	WT	WT	S	na	neg	na	S	na	-	I	32	-
**2**	10034	R	na	WT	T-A	R	na	+	na	S	na	-	I	na	+
**3**	10166	R	na	WT	T-A	R	na	+	na	S	na	-	R	na	+
**4**	21187	I	na	WT	T-A	R	na	neg	+	S	na	-	I	na	+
**5**	21330	R	4	WT	T-A	R	na	+	na	S	2	-	I	na	+
**6**	21338	I	1	WT	WT	R	na	neg	+	S	na	-	R	na	+
**7**	21347	I	4	WT	T-A	R	16	neg	+	S	na	-	I	na	+
**8**	21387	R	4	WT	WT	R	na	neg	+	R	32	aph(3′)-IIIa	S	na	-
**9**	21388	I	1	WT	WT	R	na	+	na	S	na	-	I	na	-
**10**	21390	I	4	WT	T-A	S	2	neg	na	S	na	-	R	na	+
**11**	21391	R	8	T86I	WT	R	na	+	na	I	na	-	I	na	+
**12**	24313	R	4	WT	T-A	S	na	neg	na	S	8	-	S	64	+
**13**	2110274318	R	8	T86I	T-A	R	na	+	na	S	na	-	S	16	-
**14**	2110274330	R	8	T86I	WT	R	na	+	na	S	na	-	R	na	+
**15**	2110274350	R	na	T86I	T-A	S	na	neg	na	S	na	-	R	na	+
**16**	2110274352	I	8	T86I	T-A	R	na	neg	+	S	na	-	R	na	+
**17**	2110274918	R	na	T86I	T-A	R	na	neg	+	S	na	-	R	na	+
**18**	2110278639	R	8	T86I	WT	S	na	neg	na	S	na	-	R	64	-
**19**	2110279208	R	na	T86I	T-A	R	na	+	na	S	na	-	R	na	+
**20**	2110283250	I	1	WT	WT	S	na		na	S	na	-	R	na	+
***C. coli***															
**21**	CP.70.80	S	1	WT	WT	S	na	neg	na	S	na	-	I	32	-
**22**	21057	R	8	T86I	T-C	R	na	+	na	S	na	-	R	na	+
**23**	21176	R	8	T86I	WT	R	na	neg	+	S	na	-	R	na	+
**24**	21245	R	2	WT	WT	R	na	+	na	R	16	aph(3′)-IIIa	I	64	+
**25**	21349	I	1	WT	WT	S	na	neg	na	S	na		I	na	-
**26**	24757	R	na	T86I	WT	R	na	+	na	R	32	aph(3′)-IIIaaph(2″)-If	I	na	-
**27**	2020/0011	R	na	T86I	WT	S	na	-	na	S	na	-	R	128	+
**28**	2020/0013	I	na	WT	WT	R	na	+	na	S	na	-	R	na	+
**29**	2020/0019	I	na	WT	WT	R	na	+	na	S	na	-	R	na	+
**30**	2020/0045	R	na	T86I	WT	S	na	-	na	S	na	-	I	na	-
**31**	2020/0048	R	8	T86I	WT	R	na	+	na	S	na	-	I	na	+
**32**	2020/0049	R	na	T86I	WT	R	na	+	na	S	na	-	R	na	+
**33**	2020/0073	R	na	T86I	WT	R	na	+	na	S	na	-	R	na	+
**34**	2110278602	I	na	WT	WT	R	na	neg	+	S	na	-	R	na	+
**35**	2110296031	R	na	T86I	WT	R	na	+	na	S	na	-	R	na	+
***C. fetus***															
**36**	24317	R	8	T86I	WT	R	na	+	na	S	na	-	I	na	-
**37**	24326	I	1	WT	WT	S	na	-	na	S	na	-	R	na	-
***C. lari***															
**38**	24309	I	1	WT	WT	S	na	-	na	S	2	-	R	na	-
**39**	24315	I	2	WT	WT	S	na	-	na	S	2	-	R	na	+
**40**	24316	R	4	T86V	WT	S	na	-	na	S	na	-	R	na	+
**41**	24323	R	na	T86V	WT	S	na	-	na	S	na	-	R	na	+
**42**	24324	I	4	T86V	WT	S	na	-	na	S	na	-	S	na	+
**43**	24329	R	na	T86V	WT	S	na	-	na	S	na	-	R	na	+
**44**	24756	I	na	WT	WT	S	na	-	na	S	2	-	I	na	-

^a^ Disc, agar diffusion assay; S, sensitive, I, intermediate, R, resistant. ^b^ MIC, minimum inhibitory concentration; na, not available. ^c^ WT, wild-type; T86I, threonine to isoleucine mutation at amino acid 86; T86V, threonine to valine mutation at amino acid 86. ^d^ WT, wild-type; T-A, T to A nucleotide mutation in the R box of the *cmeABC* operon. ^e^ The presence of *tet*(*O*) was assessed by next-generation sequencing (NGS) and by polymerase chain reaction (PCR); neg, not detected; +, gene detected; na, not available. ^f^ Gentamycin resistance genes detected by NGS are indicated. ^g^ The gene *bla_OXA-61_* was detected (+) or not (-) in the strains.

Only three strains were resistant to gentamycin and all carried the aminoglycoside 3′-O-phosphotransferase gene *APH* (*3*′)*-IIIa* (Table 1), except for the *C. coli* strain 24757 which also harbored a gene coding for a 2′-O-phosphotransferase (Table 1). Genome assemblies revealed that the *APH* (*3*′)*-IIIa* gene was encoded on the pTet plasmid but this was not the case for strain *C. coli* 24757 (Table S2). Only a single strain was resistant to erythromycin (*C. coli*, strain 2110296031) and we detected in its genome an A2075G mutation in the *23S rRNA* gene, a known marker of macrolide resistance in *Campylobacter* [29]. Resistance to ampicillin was reported in 90.9 percent of the strains. The most frequent β-lactam resistance gene was bla*_OXA-61_*, which was found in 15 *C. jejuni*, 11 *C. coli*, and 5 *C. lari* strains (Table 1).

### 2.2. Molecular Typing and Secondary Metabolite Biosynthesis Analysis

We used MLST to analyze our 44 isolates. Out of these, 27 STs were found to match entries in the reference pubMLST database, while 6 isolates exhibited either an untypeable locus combination or alleles that represented novel STs not present in the database. An analysis using antiSMASH showed that none of the strains harbored genes involved in the biosynthesis of bacteriocin or bacteriocin-like peptides, but we found in 12 *C. jejuni* strains a locus potentially coding for beta-lactone as a secondary metabolite (Figure S2, Table S2).

### 2.3. Chemical Synthesis and Antimicrobial Activity of the Thr1 Analog

**Figure 2:**
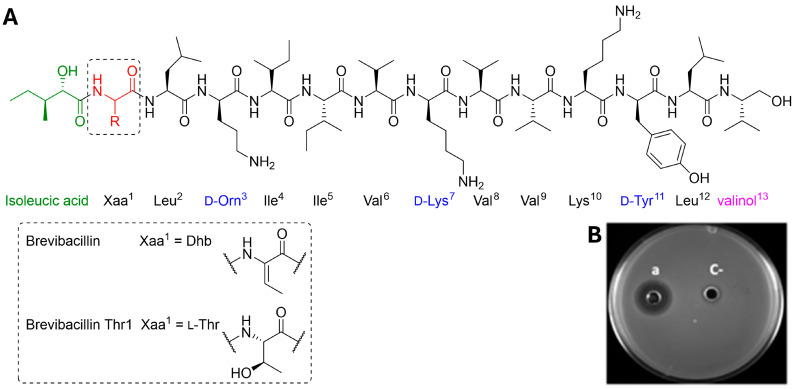
Amino acid sequence, chemical structure, and activity of the brevibacillin Thr1 analog. (**A**) The chemical structures of native brevibacillin and the brevibacillin Thr1 analog. The Thr1 analog is made of 13 amino acids, it has a molecular weight of 1602.13 Da and a net charge of +3. (**B**) Agar diffusion assay against *Campylobacter coli* CIP.70.80, (a) inhibition of bacterial growth by brevibacillin Thr1 analog (512 µg/mL) (C-) negative control 10% DMSO in water.

The lipopeptide brevibacillin contains an N-terminal l-isoleucic fatty acid and five non-proteinogenic amino acids, including three d-amino acids (d-Orn, d-Lys, d-Tyr), a C-terminal valinol and an α, β-dehydrobutyric acid (Dhb) (Figure 2A). While most of these non-canonical residues are accessible and can be used in standard solid-phase peptide synthesis, the Dhb amino acid is a lot more difficult to produce, difficult to couple during peptide elongation, and can generate side products during synthesis and handling. It was recently shown that the substitution of Dhb at position 1 of brevibacillin by threonine does not impact the antimicrobial activity and reduces hemolytic activity and cytotoxicity (Figure 2A) [30]. This modification is an important breakthrough since it greatly facilitates the synthesis of the lipopeptide and also significantly increases production yields compared to native brevibacillin. The production of the brevibacillin Thr1 analog was performed by microwave-assisted solid-phase peptide synthesis according to the protocol described by Fliss et al. [30]. The purity and identity of the analog were confirmed by high-performance liquid chromatography (HPLC) and mass spectrometry with the peptide at >95% purity and a corresponding molecular ion at 1602.13 Da (Figure S3). The antimicrobial activity of the synthetic brevibacillin Thr1 analog was first assessed against a control strain (*Campylobacter coli* CP.70.80) using the agar diffusion assay, which showed a zone of inhibition and thus qualitatively confirmed the activity of the synthetic molecule against *Campylobacter* (Figure 2B).

**Figure 3:**
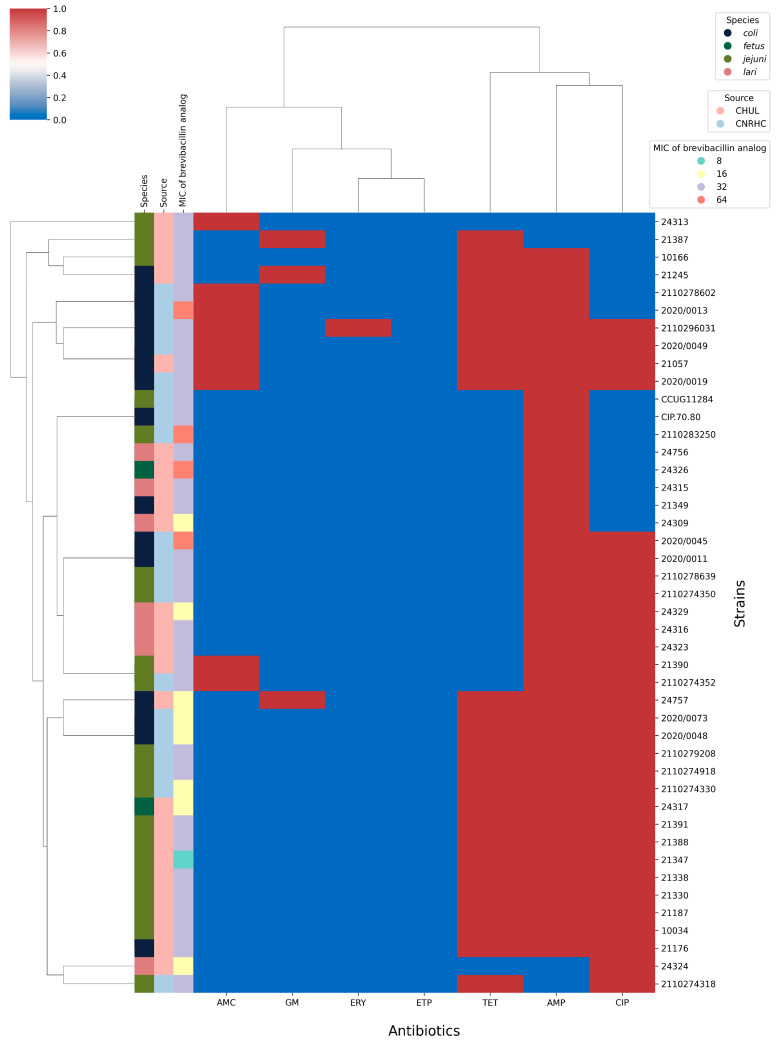
Heatmap illustrating the MIC of the brevibacillin Thr1 analog according to species and antibiotic resistance. Species and MIC values (from 8–64 µg/mL) of the brevibacillin analog are shown on the left with color codes. The activity of the brevibacillin Thr1 analog was contrasted to antibiotic susceptibility to Amoxicillin + Clavulanic Acid (AMC), Gentamycin (GMN), Erythromycin (ERY), Ertapenem (ETP), Tetracycline (TET), Amoxicillin (AMP), and Ciprofloxacin (CIP) and shown in blue (sensitive) or red (resistant).

We next tested the inhibitory activity of the brevibacillin Thr1 analog against our *Campylobacter* spp. isolates and whether resistance to antibiotics would lead to cross-resistance to the peptide. Antimicrobial activity was assessed quantitatively by determining the minimum inhibitory concentration (MIC) and minimum bactericidal concentration (MBC). The brevibacillin analog showed a heterogeneous spectrum of activities with MIC values ranging from 8 to 64 µg/mL (Figure 3). The lowest MIC observed was 8 µg/mL against one *C. jejuni.* Most strains (31/44) had a MIC of 32 µg/mL, eight strains showed a MIC of 16 µg/mL and four strains a MIC of 64 µg/mL (Figure 3). The brevibacillin Thr1 analog was found to exhibit bactericidal activity against all tested *Campylobacter* isolates as determined by their R ratio (MIC/MBC), with an R-value ≤ 4 being a hallmark of bactericidal antimicrobials (Table S3).

With the promising inhibitory activity displayed by the Thr1 analog against *Campylobacter*, we next tested whether cells can adapt to a brevibacillin challenge. To do so, two of the most sensitive strains, 21347 (MIC = 8 µg/mL) and 2110274330 (MIC = 16 µg/mL), were used and exposed to 1/4 their respective MICs. Two of the growing colonies were then passaged at 1/2 their respective parent MICs, and two colonies growing on these plates were next passaged with their respective parent MIC. However, no colonies grew at the latter concentration. Our attempt to generate brevibacillin Thr1 analog-resistant mutants was repeated but similar results were obtained, where no growth was observed when colonies derived from plates at 1/2 MIC were plated at the MIC. The MIC of cells that grew on plates in the presence of 1/2 MIC remained unchanged at 8 and 16 µg/mL.

## 3. Discussion

The Global Enteric Multicenter Study Group (GEMS) has identified *Campylobacter* as one of the primary causes of diarrhea [31] and gastrointestinal infections worldwide [32]. Antibiotic resistance is continuously growing in both human and animal cases of *Campylobacter* infection. Analysis of our strain collection confirmed the widespread occurrence of resistance and multidrug resistance in four *Campylobacter* species (Table 1). *Campylobacter* spp. is intrinsically resistant to β-lactams [33] but can gain further resistance by the acquisition of a β-lactamase gene, notably the *bla_OXA-61_* gene frequently found in *Campylobacter* [34,35]. Most strains of our collection, except four, were resistant to ampicillin (Table 1) due to *bla_OXA-61_* found in a majority of strains. Other mechanisms could also be involved such as the low ability of β-lactams to bind penicillin-binding protein (PBP) and the inability of β-lactams to penetrate the outer membrane [36]. Yet, resistance to newer generation β-lactams such as amoxicillin/clavulanic acid or ertapenem was low or inexistent in our isolates (Table S1), in concordance with previous studies [37,38].

Resistance to β-lactam antibiotics is often associated with fluoroquinolone and tetracycline resistance, leading to multidrug-resistant *Campylobacter* [39,40]. More than half of our strains (*n* = 24) were resistant to those three antibiotics (Table S1). This is in line with several previous studies [41,42,43]. We found that tetracycline resistance was mediated by *tet*(*O*) and this gene was most often encoded on a plasmid pTet [44,45]. However, in many isolates where *tet*(*O*) was detected by NGS, we failed to detect pTet (Table S4). This suggests that the short sequencing reads did not always allow the proper sequencing of the plasmid. In our initial NGS data analysis, some tetracycline-resistant *Campylobacter* isolates appeared to lack the *tet*(*O*) gene, however, we were able to detect *tet*(*O*) in these isolates by PCR (Figure S1). Possibly, *tet*(*O*) is encoded by different genetic determinants that were lost during our genome assembly and they appear to be unrelated to the pTet plasmid (Table S2).

Ciprofloxacin resistance correlated in our isolates with mutations in the GyrA target and in a *cmeR*-box controlling the expression of the CmeABC efflux pump. The mutation T86I in *gyrA* was strongly associated with ciprofloxacin resistance and is consistent with the finding of others in *C. jejuni* and *C. coli* [46,47]. Four *C. lari* isolates from our collection were resistant to ciprofloxacin. These also had a mutation in *gyrA* but it was the T86V mutation (Table 1), a mutation specific to *C. lari* [48]. Resistance to quinolones is also associated with the overexpression of the efflux pump CmeABC [48,49,50]. This pump is regulated by a repressor CmeR that binds to a 16-base-pair inverted repeat sequence (CmeR-Box) [51] (Figure 1). We found a T-C transition in one strain of *C. coli* and a T-A transversion in 13 *C. jejuni*. The same *cmeR*-box mutations have been described by others [52] and shown to lead to overexpression of the efflux pump, a significant contributor of resistance to ciprofloxacin in *Campylobacter* [53]. Our study demonstrated a good phenotype–genotype correlation and in most strains the antibiotic resistance patterns matched with the presence of a specific gene or mutations. However, this correlation in addition to the specific genes identified also stems from the activity of the multidrug-resistant efflux pump CmeABC. Indeed, overexpression of CmeABC was shown to play a prominent role in resistance to a diverse range of antibiotics, including β-lactams, fluoroquinolones, macrolides, and tetracyclines [53,54,55]. Interestingly, strain 21387, which was resistant to ciprofloxacin with a MIC of 4 mg/mL (Table 1), had no known mutation that could explain this resistance phenotype. This may be due to single-nucleotide polymorphisms that change the expression or activity of the gene product such as an efflux pump or a DNA-topology-related enzyme.

The antiSMASH tool did not reveal genes associated with bacteriocin production in our collection of *Campylobacter* strains. However, a specific locus producing a β-lactone-containing protease inhibitor was found specifically in multidrug-resistant *C. jejuni*. β-lactones were shown to inhibit proteases in bacteria, particularly the ClpP enzyme. These compounds can potentially reduce bacterial virulence by interfering with ClpP function [56]. Additionally, due to their ability to target and inhibit bacterial proteases, β-lactones are considered promising candidates for developing antimicrobial drugs [56,57,58].

Antibiotic resistance in *Campylobacter* is prevalent and antimicrobial agents with new modes of action are urgently needed. Among alternative approaches, AMPs show very promising properties and activities [59]. Among the AMPs investigated are pexiganan (a synthetic derivative of magainin), LL-37 (the only human cathelicidin), bacteriocins, which are proteinaceous toxins produced by bacteria to inhibit the growth of similar or closely related bacterial strains, and lipopeptides, which are amphipathic molecules with strong cell membrane disruption activities. These peptides have demonstrated potent antimicrobial efficacy, favorable pharmacodynamic profiles, and significant therapeutic potential [60,61]. Currently, very few reports exist in the literature specifically describing the inhibition of *Campylobacters* by AMPs. Recently, the design and chemical synthesis of a brevibacillin Thr1 analog of increased biological activity, reduced hemolytic activity, and higher production yield was reported [30]. We show here that this analog is active against a wide range of *Campylobacter* strains with MIC values between 8 µg/mL and 64 µg/mL. Importantly, the brevibacillin Thr1 analog is equally active whether the strains are sensitive or resistant to antibiotics (Figure 3). Our inability to select for resistance against this lipopeptide in *Campylobacter* is of interest and shows it has the potential to remain active despite repeated use. While the activity of brevibacillin against Gram-positive bacteria is well established [25], it is active against some Gram-negative bacteria such as *Salmonella* and *Escherichia coli* [62] and now we can add *Campylobacter*. Despite the promising antimicrobial activity of the brevibacillin Thr1 analog, further research is needed to fully assess its therapeutic potential. Key limitations include the lack of knowledge on its exact mode of action and an untested spectrum of activity against a broader range of pathogens. Structure–function activity relationships will also be important to understand the influence of secondary and tertiary structures on biological activity.Wu et al. found that brevibacillin and brevibacillin V have a greater affinity for lipopolysaccharide (LPS) in *Salmonella typhimurium*, indicating that lipopeptides can bind to LPS and displace divalent cations on the LPS network [62]. The study also demonstrated that brevibacillin and brevibacillin V can cause *S. typhimurium* to release LPS, resulting in the disruption of the dense LPS network and increased outer membrane permeability. This underlines how lipopeptides, such as brevibacillin, can target bacterial membranes. Optimization of physicochemical conditions (such as pH, ionic strength, or immobilization on supports) or synergistic interactions from its combination with other antimicrobial agents could further enhance brevibacillin’s antimicrobial activity against a wider variety of pathogens [63,64].

In summary, we have characterized a collection of human *Campylobacter* isolates at the genomic level which has allowed us to find the molecular markers of resistance. These strains are universally sensitive to the brevibacillin Thr1 analog, and this molecule could serve in our armamentarium to control *Campylobacter* and reduce the potential for antibiotic resistance.

## 4. Materials and Methods

### 4.1. Bacterial Strains

A total of 44 *Campylobacter* spp. strains were obtained from two separate collections. Nineteen strains were collected from the *Helicobacter* and *Campylobacter* Reference Center in Bordeaux, France, with the other twenty-five strains obtained from the Centre de Recherche en Infectiologie collection at Laval University. Most strains were isolated from human clinical samples (*n* = 42), whereas two reference strains were initially isolated from animals. The collection included *Campylobacter jejuni* (*n* = 20), *Campylobacter coli* (*n* = 15), *Campylobacter lari* (*n* = 7), and *Campylobacter fetus* (*n* = 2). Details regarding the strains can be found in Table S4.

### 4.2. Campylobacter Culture

*Campylobacter* spp. was cultivated on blood agar (Becton Dickinson & Co., One Becton Drive, Franklin Lakes, NJ, USA) supplemented with 5% lysed horse blood under microaerophilic conditions (5% O_2_, 10% CO_2_, 85% nitrogen, and >80% humidity), using *Campylobacter* gas pack systems (BD GasPak™ EZ Anaerobe Container System, Becton Dickinson & Co., One Becton Drive, Franklin Lakes, NJ, USA), at 37 °C or 42 °C depending on the species (*C. coli* and *C. jejuni* at 42 °C, *C. lari* and *C. fetus* at 37 °C) for 48 h.

### 4.3. Antimicrobial Susceptibility Test

The standard agar disc diffusion assay was used to evaluate the antimicrobial susceptibility of our *Campylobacter* strains following the Clinical and Laboratory Standards Institute (CLSI, 2020) and the European Committee on Antimicrobial Susceptibility Testing (EUCAST, 2021) guidelines [65,66]. The following seven antibiotic discs were used: amoxicillin (AMP) (10 μg), amoxicillin + clavulanic acid (AMC) (20/10 μg), ertapenem (ETP) (10 μg), erythromycin (ERY) (15 μg), gentamycin (GM) (10 μg), tetracycline (TET) (30 μg), and ciprofloxacin (CIP) (5 μg). After 48 h of microaerophilic incubation at 37 °C, the diameter of the zone of inhibition surrounding antibiotic discs was measured and translated into sensitive (S), intermediate (I), and resistant (R) categories according to CLSI and EUCAST guidelines. According to the EUCAST definition, ‘intermediate’ does not equate to resistance. Therefore, the presented values refer to isolates classified as both ‘intermediate’ (I) and ‘resistant’ (R). Minimum inhibitory concentrations by microdilution were made for selected strains to confirm the observed phenotype when strains exhibited an intermediate phenotype or when phenotypes did not align with genotypes.

### 4.4. Whole-Genome Sequencing and Data Analysis

Genomic DNA was extracted using a Wizard^®^ Genomic DNA Purification Kit (Promega, Woods Hollow Road, Madison, WI, USA). Libraries were prepared using the Illumina DNA Prep kit according to the manufacturer’s instructions (Illumina, Illumina Way, San Diego, CA, USA). Sequencing libraries were quantified using the QuantiFluor dsDNA System, and their size distribution was validated using a TapeStation system (Agilent, Stevens Creek Blvd. Santa Clara, CA, USA). Paired-end sequencing was performed on an Illumina MiSeq platform. Sequencing reads were trimmed according to their base quality using the Trimmomatic version 0.39 software. Draft genomes were de novo assembled using SPAdes version 3.15.1 with default settings. The sequence type (ST) of the strains was determined from the contigs using the MLST software version 2.23.0 [67]. The analysis of the *Campylobacter* spp. allele scheme was set from the open-access PubMLST [68]. PlasmidSPAdes version 3.15.4, with default settings, was employed to execute de novo plasmid assembly with raw read error correction [69]. Additionally, the presence of established plasmids was assessed by performing reference-based mapping through PlasmidSeeker [70]. The Comprehensive Antibiotic Resistance Database (CARD) [71] and the Pathosystems Resource Integration Center (PATRIC) [72] were used for searching for antibiotic resistance markers. Prediction of secondary metabolite biosynthesis gene clusters from contig sequences was performed using Antibiotics & Secondary Metabolite Analysis Shell (antiSMASH) version 6 with default parameters [73]. Sequence Read Archive biosample accessions for the samples sequenced in this study can be found in Table S5.

### 4.5. Production of the Brevibacillin Thr1 Analog

The brevibacillin Thr1 analog was prepared by standard solid-phase peptide synthesis (SPPS) using the Fmoc/tBu strategy on a preloaded valinol-Wang polystyrene resin (typically 0.3 mmol/g) as previously described [30]. After cleavage from the resin and side chains’ deprotection with a solution containing trifluoroacetic acid, triisopropylsilane, and water (TFA/TIS/H_2_O, 95:2.5:2.5), the resulting peptide was precipitated in cold diethyl ether and the solid dried under vacuum. The crude peptide was purified to >95% homogeneity by semi-preparative RP-HPLC with a Shimadzu Prominence system on a Phenomenex Kinetex EVOC18 column (250 mm × 21.2 mm, 300 Å, 5 μm) using 0.1%TFA/H_2_O (A) and 0.1% TFA/CH_3_CN (B), with a linear gradient from 10 to 60% (B) for 20 min at a rate of 12 mL/min and UV detection at 220 and 254 nm. The collected fractions were lyophilized to afford the desired brevibacillin Thr1 analog as a white powder.

### 4.6. Antimicrobial Activity of the Brevibacillin Analog

The antimicrobial activity of the brevibacillin Thr1 analog was evaluated by agar diffusion and broth microdilution against the 44 clinical *Campylobacter* spp. strains in triplicates. For agar dilution, bacteria were cultured in Brucella broth medium (BBL™ Difco™, One Becton Drive, Franklin Lakes, NJ, USA) at 37–42 °C for 48 h to promote optimal growth. After the incubation period, 1% of inoculum was prepared and added to Brucella soft agar medium (0.75%). Wells were carefully created in the agar and filled with a precisely measured volume of 80 μL of peptide at a concentration of 512 μg/mL. The plates were then incubated under microaerophilic conditions for 48 h either at 37 or 42 °C to allow for bacterial growth and potential inhibition or antimicrobial effects to be observed. The MIC of brevibacillin was determined by broth microdilution in 96-well microtiter plates and according to the CLSI guidelines regarding inoculum density, growth medium, incubation time, and conditions. For these experiments, isolated colonies obtained from overnight cultures were suspended in phosphate-buffered saline (PBS) and adjusted to turbidity corresponding to the McFarland standard of 0.5. A volume of 50 µL of bacterial suspension was added to each well of the microtiter plate, along with 125 μL of brevibacillin analog (ranging from 512 µg/mL to 1 µg/mL diluted in series) and 125 µL of growth medium. The microplates were incubated for 24–48 h, within a temperature range of 37–42 °C, under microaerophilic conditions. The MIC was determined as the lowest brevibacillin concentration that completely inhibited bacterial growth. The MBC was determined by inoculating MH agar supplemented with 5% lysed horse blood with 10 µL from wells showing complete growth inhibition followed by incubation for 48 h within a temperature range of 37–42 °C under microaerophilic conditions. Reference strains *C. jejuni* (CIP.70.80) and *C. coli* (CCUG1184) were included in the experiments and served as references for evaluating the antimicrobial activity and validating the accuracy and reliability of the assays.

### 4.7. Mutants’ Selection

To examine whether *Campylobacter* can be selected for resistance to the brevibacillin analog we chose two susceptible strains (21347 and 2110274330) that were serially exposed to increasing concentrations of brevibacillin analog. Two strains were then started from 48 h cultures from independent colonies and then were serially replicated in 1 ml cultures every 48–72 h in Brucella broth medium containing the synthetic peptide. The susceptibility of the selected strains was verified by microdilution assay.

### 4.8. Statistical Analysis

Antimicrobial resistance genes were considered as a binary dependent variable (0 = not detected; 1 = detected). Data were analyzed using SPSS version 20 software (IBM Corporation, Somers, NY, USA) applying Pearson’s chi-square test. The level of statistical significance was set at *p* < 0.05. A heatmap was made using the clustermap function from the seaborn package (v0.13.1) [74].

## Data Availability

Sequence reads from this project have been deposited in the Sequence Read Archive (https://www.ncbi.nlm.nih.gov/sra) under biosample accessions listed in Table S5.

## Supplementary Materials

The following supporting information can be downloaded at: https://www.mdpi.com/article/10.3390/ijms26104657/s1.
