# Supplementary material for: Antimicrobial Activity of a Synthetic Brevibacillin Analog Against Multidrug-Resistant Campylobacter spp
Source: Int J Mol Sci. 2025 May 13;26(10):4657. doi: 10.3390/ijms26104657 (PMC12111383; doi:10.3390/ijms26104657)
Supplement: Supplementary file 1 [file ijms-26-04657-s001.zip › ijms-3568970-supplementary.pdf]

International Journal of
Molecular Sciences

MDPI

Supplementary Materials to Manuscript

Antimicrobial Activity of a Synthetic Brevibacillin Analog Against Multidrug-Resistant *Campylobacter* spp.

Khaled Abdallah, Omar Fliss, Nguyen Phuong Pham, Louis David Guay, Ghassan Tayh, Hélène Gingras, Chantal Godin, Phillippe Leprohon, Eric Biron, Ismail Fliss and Marc Ouellette

Table S1. Antimicrobial resistance rates among the investigated *Campylobacter* spp.

	***Campylobacter* spp. (n = 44)**			
	**Susceptible (S)**	**Intermediate (I)**	**Resistant (R)**	**Non-susceptible (I+R)**
	**no. (%)**			
**Ampicillin**	4(9.1)	15(34.1)	25(56.8)	40(90.9)
**Amoxicillin + Clavulanic Acid**	34(77.3)	7(15.9)	3(6.8)	10(22.7)
**Erythromycin**	43(97.7)	0(0)	1(2.3)	1(2.3)
**Tetracycline**	18(40.9)	0(0)	26(59.1)	26(59.1)
**Ciprofloxacin**	13(29.5)	4(9.1)	27(61.4)	31(70.45)
**Gentamycin**	41(93.2)	0(0)	3(6.8)	3(6.8)
**Ertapenem**	44(100)	0(0)	0(0)	0(0)

Table S2. Identified plasmids and secondary metabolite production in selected *Campylobacter* strains.

**Strains**	***Tet(O)***	**Other genes**	**pTet**	**RefSeq sequence**	**Betalactone production**	**STs**
C. jejuni						
**CCUG11284**	-	---	0	---	-	403
**10034**	+	---	1	NZ_CP045046.1	+	50
**10166**	+	---	1	NZ_CP010302.1	-	9506
**21187**	-	---	0	---	+	21
**21330**	+	---	1	NZ_CP007750.1	-	1244
**21338**	-	---	0	---	+	2085
**21347**	-	---	0	---	-	New ST
**21387**	-	APH(3')-IIIa	0	---	+	1035
**21388**	+	---	1	NZ_CP007750.1	-	459
**21390**	-	---	0	---	+	21
**21391**	+	---	0	---	-	1244
**24313**	-	---	0	---	+	21
**2110274318**	+	---	0	---	-	122
**2110274330**	+	---	0	---	+	354
**2110274350**	-	---	0	---	-	19
**2110274352**	-	---	0	---	-	19
**2110274918**	-	---	0	---	+	122
**2110278639**	-	---	0	---		19
**2110279208**	+	---	1	NZ_CP011017	+	New ST
**2110283250**	-	---	0	---	+	45

C. coli						
**CIP.70.80**	-	---	0	---	-	900
**21057**	+	---	1	NZ_CP017869.1	-	825
**21176**	-	---	-	---	-	827
**21245**	+	APH(3′)-IIIa	1	NC_022354.1	-	1068
**21349**	-	---	0	---	-	829
**24757**	+	APH(3′)-IIIa<br>APH(2′)-IF	1<br>0	NZ_CP013035.1<br>---	-	825
**2020/0011**	-	---	0	---	-	827
**2020/0013**	+	---	0	---	-	1595
**2020/0019**	+	---	0	---	-	860
**2020/0045**	-	---	0	---	-	1055
**2020/0048**	+	---	0	---	-	832
**2020/0049**	+	---	0	---	-	1666
**2020/0073**	+	---	0	---	-	827
**2110278602**	-	---	0	---	-	1243
**2110296031**	+	---	0	---	-	832
C. fetus						
**24326**	-	---	0	---	-	6
**24317**	+	---	1	NZ_CP013035.1	-	3
C. lari						
**24309**	-	---	0	---	-	New ST
**24315**	-	---	0	---	-	New ST
**24316**	-	---	0	---	-	New ST
**24323**	-	---	0	---	-	8
**24324**	-	---	0	---	-	37
**24329**	-	---	0	---	-	37
**24756**	-	---	0	---	-	New ST

-: not detected, +: detected, ---: no gene or no plasmid reference, 0: plasmid not detected, 1: plasmid detected, STs: sequence type.

Table S3. MIC, MBC Values, and MBC/MIC Ratio of brevibacillin analog against *Campylobacter* spp.

**Strains**	**Concentration (µg.ml-1)**			**Effect**
	MIC	MBC	Ratio MBC/MIC	Antimicrobial effect
C. jejuni				
**CCUG11284**	32	32	1	Bactericidal
**10034**	32	32	1	Bactericidal
**10166**	32	32	1	Bactericidal
**21187**	32	64	2	Bactericidal
**21330**	32	32	1	Bactericidal
**21338**	32	32	1	Bactericidal
**21347**	8	8	1	Bactericidal
**21387**	32	64	2	Bactericidal
**21388**	32	32	1	Bactericidal
**21390**	32	32	1	Bactericidal
**21391**	32	32	1	Bactericidal
**24313**	32	64	2	Bactericidal
**2110274318**	32	32	1	Bactericidal
**2110274330**	16	16	1	Bactericidal
**2110274350**	32	32	1	Bactericidal

**2110274352**	32	32	1	Bactericidal
**2110274918**	32	32	1	Bactericidal
**2110278639**	32	32	1	Bactericidal
**2110279208**	32	32	1	Bactericidal
**2110283250**	64	128	1	Bactericidal
C. coli				
**CIP.70.80**	32	32	1	Bactericidal
**21057**	32	32	1	Bactericidal
**21176**	32	32	1	Bactericidal
**21245**	32	32	1	Bactericidal
**21349**	32	32	1	Bactericidal
**24757**	16	32	2	Bactericidal
**2020/0011**	32	32	1	Bactericidal
**2020/0013**	64	64	1	Bactericidal
**2020/0019**	32	32	1	Bactericidal
**2020/0045**	64	64	1	Bactericidal
**2020/0048**	16	16	1	Bactericidal
**2020/0049**	32	64	2	Bactericidal
**2020/0073**	16	16	1	Bactericidal
**2110278602**	32	64	2	Bactericidal
**2110296031**	32	32	1	Bactericidal
C. fetus				
**24326**	64	128	2	Bactericidal
**24317**	16	16	1	Bactericidal
C. lari				
**24309**	16	16	1	Bactericidal
**24315**	32	32	1	Bactericidal
**24316**	32	32	1	Bactericidal
**24323**	32	32	1	Bactericidal
**24324**	16	16	1	Bactericidal
**24329**	16	32	2	Bactericidal
**24756**	32	32	1	Bactericidal

Table S4. Characteristics of the studied strains.

Year of isolation	Origin	*C. coli* (n=15)	*C. jejuni* (n=20)	*C. fetus* (n=2)	*C. lari* (n=7)
**1981**	Animals	1	1	0	0
**2010**	Human	4	6	0	1
**2011**	Human	2	10	0	0
**2013**	Human	0	0	0	1
**2014**	Human	1	0	0	2
**2017**	Human	0	0	2	3
**2019**	Human	0	1	0	0
**2020**	Human	7	0	0	0
**Unknow**	Human	0	2	0	0

Table S5. Sequence and accession numbers for the *Campylobacter* strains sequenced in this study.

Accession	Sample Name	Organism	Taxon ID
SAMN40911393	10034	*Campylobacter jejuni*	197
SAMN40911394	10166	*Campylobacter jejuni*	197

SAMN40911395	2020-0013	*Campylobacter coli*	195
SAMN40911396	2020-0019	*Campylobacter coli*	195
SAMN40911397	2020-0045	*Campylobacter coli*	195
SAMN40911398	2020-0048	*Campylobacter coli*	195
SAMN40911399	2020-0049	*Campylobacter coli*	195
SAMN40911400	2020-0073	*Campylobacter coli*	195
SAMN40911401	21057	*Campylobacter coli*	195
SAMN40911402	2110274318	*Campylobacter jejuni*	197
SAMN40911403	2110274330	*Campylobacter jejuni*	197
SAMN40911404	2110274350	*Campylobacter jejuni*	197
SAMN40911405	2110274352	*Campylobacter jejuni*	197
SAMN40911406	2110274918	*Campylobacter jejuni*	197
SAMN40911407	2110278602	*Campylobacter coli*	195
SAMN40911408	2110278639	*Campylobacter jejuni*	197
SAMN40911409	2110279208	*Campylobacter jejuni*	197
SAMN40911410	2110283250	*Campylobacter jejuni*	197
SAMN40911411	2110296031	*Campylobacter coli*	195
SAMN40911412	21176	*Campylobacter coli*	195
SAMN40911413	21187	*Campylobacter jejuni*	197
SAMN40911414	21245	*Campylobacter coli*	195
SAMN40911415	21330	*Campylobacter jejuni*	197
SAMN40911416	21338	*Campylobacter jejuni*	197
SAMN40911417	21347	*Campylobacter jejuni*	197
SAMN40911418	21349	*Campylobacter coli*	195
SAMN40911419	21387	*Campylobacter jejuni*	197
SAMN40911420	21388	*Campylobacter jejuni*	197
SAMN40911421	21390	*Campylobacter jejuni*	197
SAMN40911422	21391	*Campylobacter jejuni*	197
SAMN40911423	24309	*Campylobacter lari*	201
SAMN40911424	24313	*Campylobacter jejuni*	197
SAMN40911425	24315	*Campylobacter lari*	201
SAMN40911426	24316	*Campylobacter lari*	201
SAMN40911427	24317	*Campylobacter fetus*	196
SAMN40911428	24323	*Campylobacter lari*	201
SAMN40911429	24324	*Campylobacter lari*	201
SAMN40911430	24326	*Campylobacter fetus*	196
SAMN40911431	24329	*Campylobacter lari*	201
SAMN40911432	24756	*Campylobacter lari*	201
SAMN40911433	24757	*Campylobacter coli*	195
SAMN40911434	CP-70-80	*Campylobacter coli*	195
SAMN40911435	CCUG11284	*Campylobacter jejuni*	197
SAMN40911436	2020-0011	*Campylobacter coli*	195

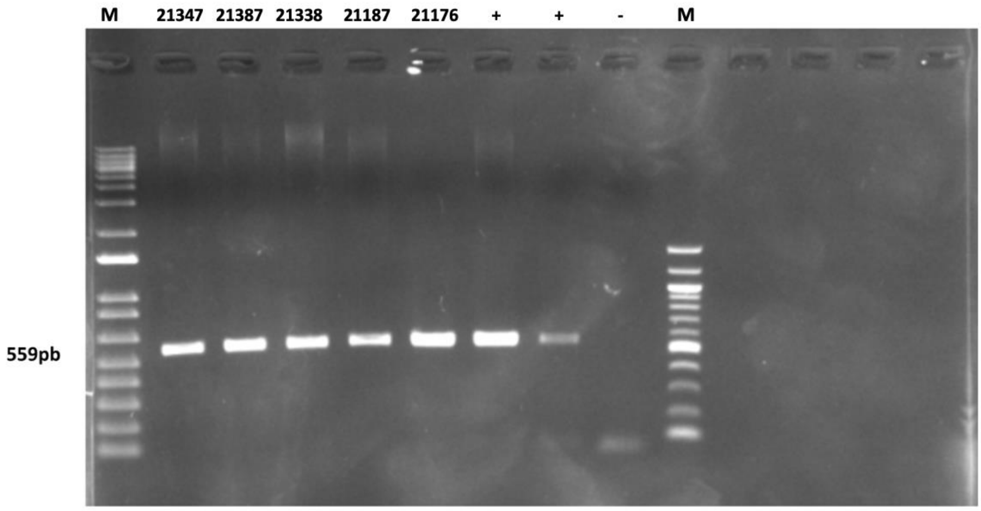

Figure S1. PCR detection of *tet*(O) from *Campylobacter spp*. strains.

DNA samples were amplified with specific primers (*tet(O))* and then evaluated using 1% agarose gel electrophoresis. The PCR conditions for *tet(O*) amplification was as follows: 2 minutes at 98°C, 35 cycles consisting of 2 min at 98°C, 30s at 53°C, 20s at 72°C, and a final extension step of 7 minutes at 72°C.

M: DNA ladders, Five *Campylobacter* strains 21347, 21387, 21338, 21187, and 21176, that were resistant to tetracycline but where *tet(O)* was not detected by NGS (see Table 1), it was detected here by PCR. +: positive controls strains 21057 and 10034 in which *tet(O)* was detected by NGS, -: negative control (Nuclease free water). Additional PCR reactions have shown amplification of the tetO genes in three tetracycline resistant strains 2110274352, 2110274918, 2110278602 for which the *tet(O*) gene was not detected by NGS. The primers used for amplification were: F: 5′-GGCGTTTTGTTTATGTGCG-3′, R: 5′-ATGGACAACCCGACAGAAGC-3′.

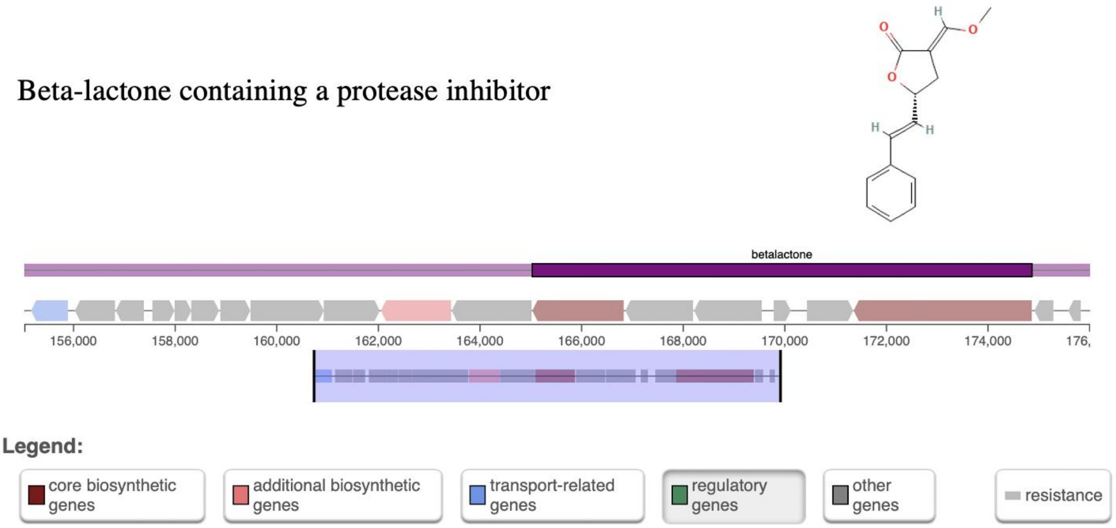

Figure S2. Operon for biosynthesis of the beta-lactone inhibitor of *Campylobacter jejuni.* (https://antismash.secondarymetabolites.org)

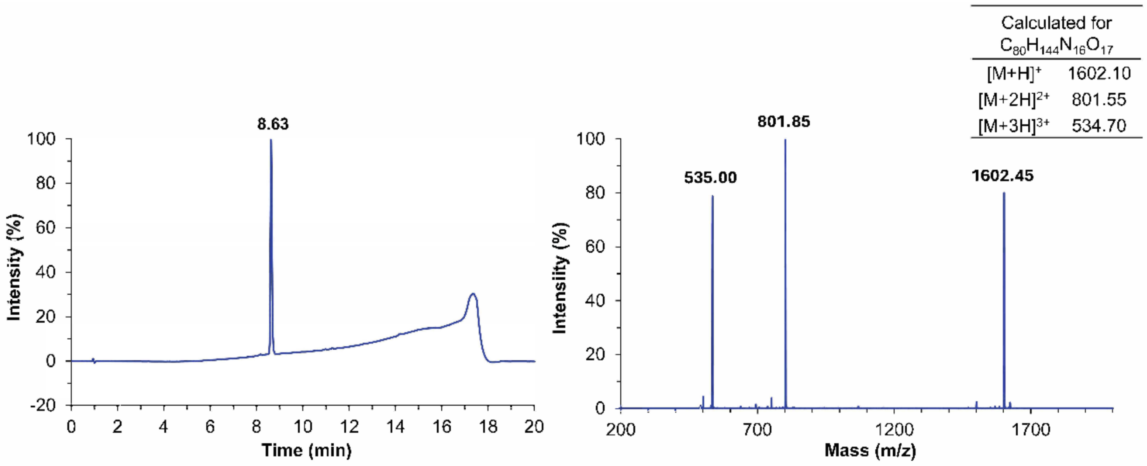

Brevibacillin Thr1 : Isolated as a white powder. R_t = 8.63 min;
HRMS (ESI+): calcd for $C_{80}H_{144}N_{16}O_{17}$ $[M + H]^+$ 1602.0968 m/z, found 1602.1031 m/z, $[M+2H]^{2+}$ 801.5520 m/z, found 801.5512 m/z.

Figure S3. HPLC profiles (λ = 220 nm) and ESI-MS spectra of brevibacillin Thr1.
